# Supplementary material for: Normalising the Implementation of Pharmacogenomic (PGx) Testing in Adult Mental Health Settings: A Theory-Based Systematic Review
Source: J Pers Med. 2024 Sep 27;14(10):1032. doi: 10.3390/jpm14101032 (PMC11508855; doi:10.3390/jpm14101032)
Supplement: Supplementary file 1 [file jpm-14-01032-s001.zip › jpm-3184826-supplementary/Zip file to upload/Supplementary Material S1.pdf]

Supplementary Material S1: Summary of Data Synthesis using Thematic Analysis and NPT

Stage 1 – Example of mind mapping raw barrier/facilitators to enable data familiarisation

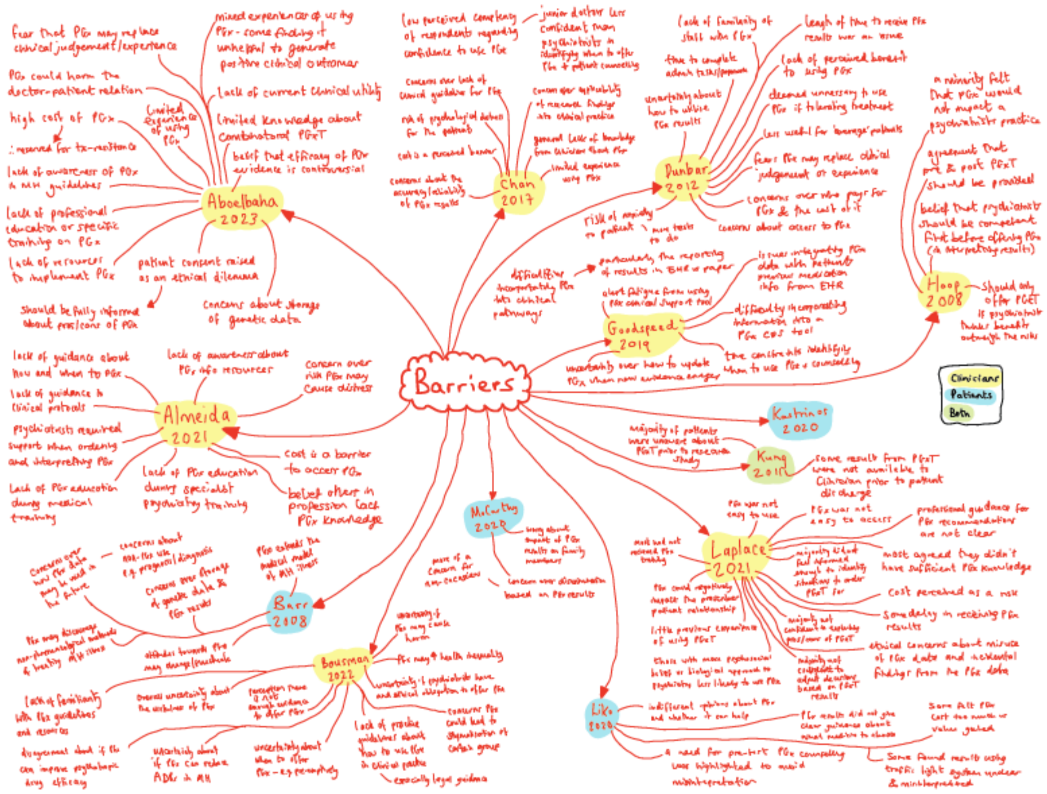

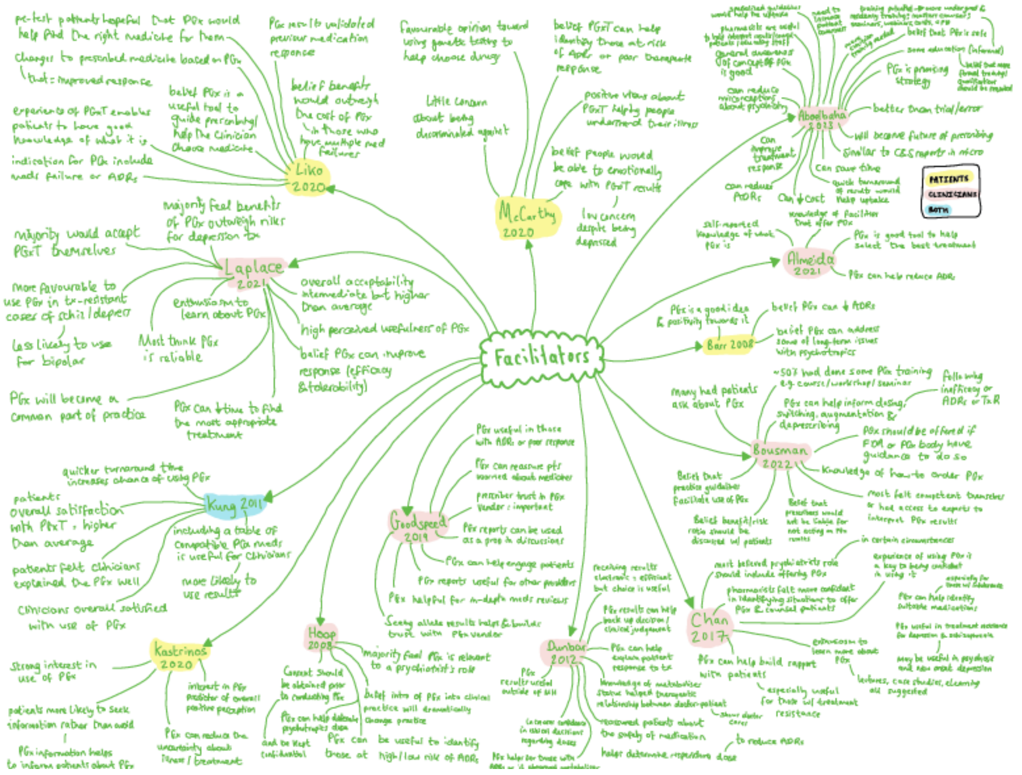

Stage 2 – Example of tabulation of raw barrier/facilitators to sub-constructs of the NPT coding framework

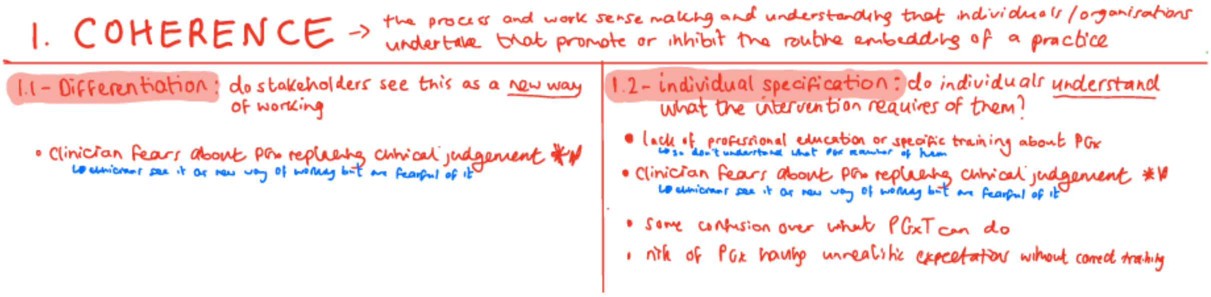

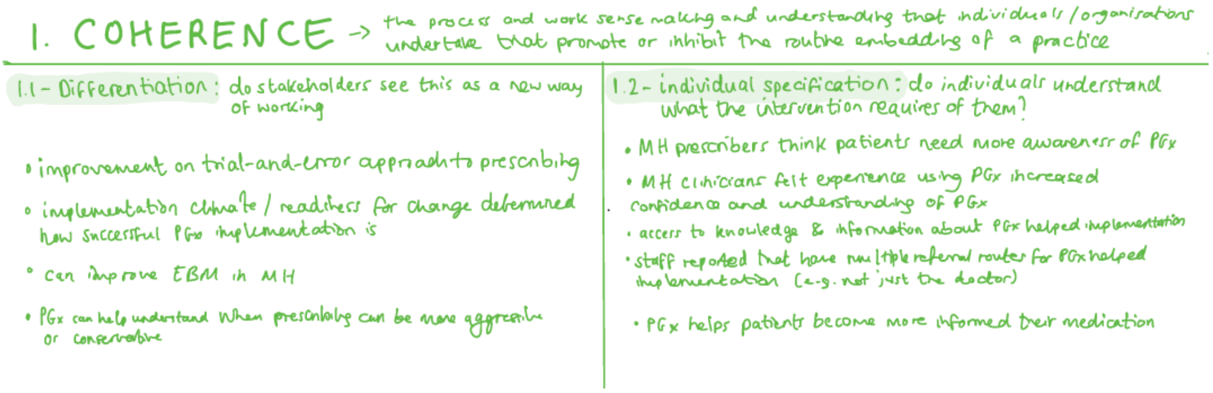

Stage 3 – Example of summarising of barriers/facilitators within sub-constructs of NPT

1. COHERENCE → the process and work sense making and understanding that individuals / organisations undertake that promote or inhibit the routine embedding of a practice

1.1 - Differentiation: do stakeholders see this as a new way of working

- Some patients see PGxT as an extension of the medical model of mental illness that already exists
- Clinicians do see PGxT as a new way of working, but some perceive that it may replace existing ways of working by replacing clinical judgement (rather than complement it)

1.2 - individual specification: do individuals understand what the intervention requires of them?

- Some confusion / uncertainty about what PGxT requires from clinicians and general perception there is a lack of understanding about PGxT and that some may have unrealistic expectations of what PGxT can do.
- Some patients did not understand what PGxT involves for them as they were not aware of what PGxT is.
- Patients understanding of what PGxT requires of them may change / fluctuate with time.

1. COHERENCE → the process and work sense making and understanding that individuals / organisations undertake that promote or inhibit the routine embedding of a practice

1.1 - Differentiation: do stakeholders see this as a new way of working

Stakeholders – in particular clinicians and predominantly psychiatrists, see PGx-guided psychotropic prescribing as a new approach to prescribing that is an improvement on the currently adopted trial-and-error approach that can build on evidence-based medicine in MH

1.2 - individual specification: do individuals understand what the intervention requires of them?

Although there are multiple models of adopting PGx testing in practice, clinician stakeholders do understand what PGxT requires of them. This appears more evident when education & training has been provided, and in those who have experience of using PGxT.

Regarding patients, prior to involvement in research studies, understanding of what PGxT requires of them was generally poor and points towards more education & awareness needed for patients

→ this more of a barrier for patients

Stage 4 – Example of summarising of barriers/facilitators within sub-constructs of NPT

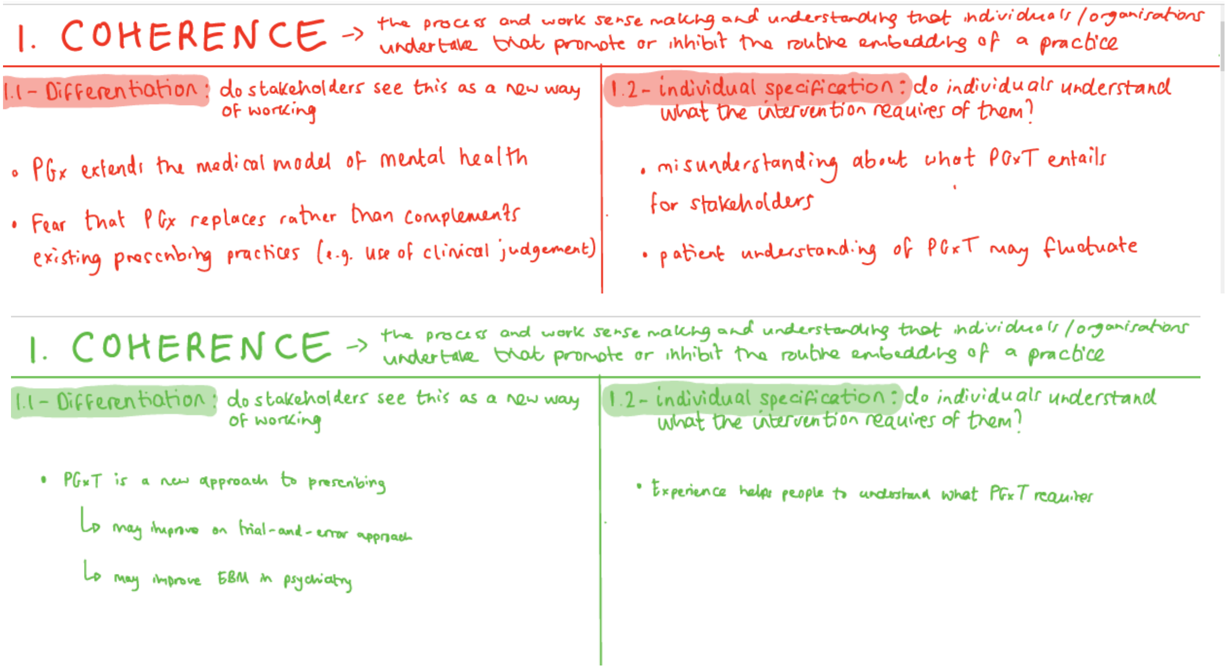

Stage 5 – Broad theme construction across NPT constructs
