# Supplementary material for: Normalising the Implementation of Pharmacogenomic (PGx) Testing in Adult Mental Health Settings: A Theory-Based Systematic Review
Source: J Pers Med. 2024 Sep 27;14(10):1032. doi: 10.3390/jpm14101032 (PMC11508855; doi:10.3390/jpm14101032)
Supplement: Supplementary file 1 [file jpm-14-01032-s001.zip › jpm-3184826-supplementary/Zip file to upload/Supplementary Material S2.pdf]

Supplementary Material S2: QuADS Tool Score, Barriers, Facilitators, Strengths, and Limitations of Individual Included Studies

Title, Author & Year	QuADS Tool Score	Barriers	Facilitators	Strengths	Limitations
**Mental Health Prescribers' Perceptions on the Use of Pharmacogenetic Testing in the Management of Depression in the Middle East and North Africa Region**<br><br>**Aboelbaha, 2023**	23	Fear PGx may replace clinical judgement or disregard experience<br><br>Belief PGx could harm doctor-patient relationship<br><br>Perceived high cost of PGx<br><br>Lack of awareness of psychiatry PGx guidelines<br><br>Lack of professional education or specific training about PGx<br><br>Lack of resource to implement PGx<br><br>Patient consent & counselling raised as an ethical problem<br><br>Concerns over storage of genetic data<br><br>Belief PGx efficacy is controversial and currently lacks clinical utility<br><br>Mixed experiences using PGx with some finding it unhelpful to generate positive clinical outcomes	Belief PGx can improve treatment response, reduce ADRs, reduce healthcare costs, reduce misconceptions in psychiatry, can save time finding suitable treatment, is a promising new strategy and is better than trial-and-error prescribing approach<br><br>Belief that a quick turnaround of PGx results, plus specialised psychiatry PGx guidelines would help uptake, and perception PGx will become the future of prescribing<br><br>Belief PGx is safe and a similar concept to use of culture/sensitivity reports in microbiology<br><br>General awareness about the concept of PGx<br><br>A desire amongst prescribers for more basic and specialised training, both informal (seminars, webinars, conferences & CPD) and formal with some believing specific training or a qualification (e.g., a master's degree) should be required to utilise PGx<br><br>Role of pharmacists perceived to be useful in PGx counselling for patients, interpreting PGx results, and educating staff	First study to assess prescriber's perspectives about PGx in the Middle East and North Africa region<br><br>Study had a wide reach in the range of countries it recruited participants from<br><br>Validated findings from previous studies exploring the use of PGx to support depression, but also generated novel ideas within the field	Generalisability of the results are limited to psychiatrists only, yet PGx is a multidisciplinary field and will require other professions input for successful implementation<br><br>Psychiatrists are not the only HCPs that prescribe antidepressants, so the views of non-psychiatrists (e.g., GPs) was missing<br><br>For many of included countries in the study there was only one participant per country, therefore the wider generalisability about perspectives from specific countries is limited<br><br>Potential that interviewer bias occurred due to qualitative nature of the study

**Perception and knowledge of pharmacogenetics among Brazilian psychiatrists**<br><br>**Almeida, 2021**	29	Lack of clinical protocols / guidance about how and when to offer PGx<br><br>Psychiatrists needed support to offer and order PGx<br><br>Lack of PGx education during medical training and during specialist psychiatry training<br><br>Awareness of PGx information resources was minimal<br><br>Concern PGx may cause psychological distress to patients<br><br>Concerns about confidentiality of PGx<br><br>Cost perceived to be a barrier to access<br><br>Belief that other psychiatrists in the profession lack the knowledge needed to offer and use PGx	Some participants reported self-knowledge of what PGx is<br><br>Belief that PGx is a good tool to help select the best treatment<br><br>Perception that PGx can help reduce ADRs<br><br>Reported that having PGx tests in the list of procedures available from the National Supplementary Health Agency and knowledge of the local facilities that offer PGx is helpful	First study of its kind to assess psychiatrist's perspectives about PGx in Brazil<br><br>Wide reaching recruitment gave a representative sample from psychiatrists across Brazil<br><br>Validated findings from previous studies conducted with psychiatrists in other countries<br><br>May act as a preliminary analysis for further investigation involving a larger number of psychiatrists from a more diverse range of geographical locations in Brazil<br><br>Findings may encourage development of PGx educational & training resources	Generalisability of the results are limited due to the use of non-probability sampling as the use of snowball sampling may have meant the questionnaire was shared amongst specific networks of psychiatrists with certain views on PGx<br><br>Small sample size
**The great ambivalence: factors likely to affect service user and public acceptability of the pharmacogenomics of antidepressant medication**<br><br>**Barr, 2008**	19.5	Concerns about the use of genetic data from PGx testing – worries it may be used in a non-PGx context e.g., in prognosis or diagnosis<br><br>Concerns about the storage of genetic data & PGx results	Belief that PGx is a good idea and general positivity towards it from patients<br><br>The polish focus group believed PGx to be a futuristic technology<br><br>Perception that PGx can help to reduce ADRs	First study of its kind to explore patient perspectives towards the use of PGx for antidepressant prescribing<br><br>Findings suggest there are several implications for emerging medical technologies	Findings of the study limited by the fact that many participants had not heard of pharmacogenomics before participating in the study therefore may not have been informed enough about PGx to answer questions<br><br>Lack of clarity about recruitment and sample size, and unclear what

		Belief that PGx extends the medical model of treating mental health illness<br><br>Perception PGx may discourage non-pharmacological methods of treating mental health illness<br><br>Belief that patient attitudes towards PGx may change or fluctuate	Belief that PGx can help address some long-term issues associated with psychotropics		the eligibility criteria to participate in the study was<br><br>Detailed comparison between nations was lacking
**Perspectives on the Clinical Use of Pharmacogenetic Testing in Late-Life Mental Healthcare: A Survey of the American Association of Geriatric Psychiatry Membership**<br><br>**Bousman, 2022**	23.5	Uncertainty about whether PGx may cause harm & if it could increase health inequalities due to ability to pay for PGx testing<br><br>Uncertainty about when to offer PGx & psychiatrists unsure if they have an ethical obligation to offer PGx<br><br>Belief there is a lack of practice guidelines for the use of PGx, including legal guidance<br><br>Perception PGx could stigmatise certain groups<br><br>Lack of consensus about whether PGx can reduce ADRs or improve psychotropic efficacy<br><br>Perception there is not enough evidence to offer PGx and overall uncertainty about PGx usefulness	Many reported patients interest in PGx<br><br>Around half of participants had received some PGx training (e.g., course, workshop, seminar)<br><br>Belief PGx can help inform dosing, switching, augmentation, and deprescribing of psychotropics, particularly following drug inefficacy, ADRs or treatment resistance<br><br>Perception that if there is FDA or PGx body guidance to offer PGx it should be used, and that practice guidelines would facilitate PGx use<br><br>Prescribers believed they would not be liable for not acting on PGx results<br><br>Many felt competent to order and utilise PGx, or had access to expertise to help	First study to explore perspectives about the use of PGx specifically in geriatric psychiatry<br><br>First study of its kind to explore whether geographical region as a potential factor that may influence attitudes towards PGx	Small sample size limits the generalisability of the results, therefore the findings may not represent all members of the American Association of Geriatric Psychiatry Membership<br><br>The questions did not ask about specific PGx tests available to participants, as it is known there is some variability in the genes/alleles tested between different PGx tests

		Lack familiarity with PGx guidance & resources	Belief the benefits/risks of PGx should be discussed with patients		
**Clinician's perceptions of pharmacogenomics use in psychiatry**<br><br>**Chan, 2017**	30.5	Concerns about the accuracy or reliability of PGx results<br><br>Perceived lack of clinical guidelines for PGx use<br><br>Belief there is a risk of psychological distress for patients receiving PGx<br><br>Cost is perceived to be a barrier to PGx use<br><br>Respondents had a low perceived competency & confidence to use PGx – females & junior doctors (compared to psychiatrists) were less confident in identifying when to offer PGx and providing PGx counselling.<br>Those less confident in ordering PGx were more concerned about clinical guidelines for PGx, and those who were less confident in making treatment recommendations based on PGx were more concerned about causing psychological distress<br><br>Clinicians reported a general lack of knowledge about PGx and limited experience using PGx	A belief that psychiatrists role should include offering PGx in certain circumstances<br><br>Pharmacists felt more confident in identifying situations to offer PGx & counsel patients<br><br>Experience of using PGx increased confidence using PGx<br><br>Belief that PGx can help build rapport with patients and is especially useful in those with treatment resistance<br><br>Perception PGx can help identify suitable medications – particularly in treatment resistance<br>Enthusiasm amongst clinicians to learn more about PGx – lectures, case studies, & e-learning all suggested as methods for education<br><br>Belief PGx is useful in treatment resistance in schizophrenia & depression<br><br>Hope for the future of PGx and belief there is opportunity for further development	Study was able to find gender and experience/seniority differences in responses to questions, particularly around perceived competency & risks<br><br>First study of its kind in the Asia region<br><br>First study of its kind to explore perceptions of both psychiatrists and pharmacists simultaneously<br><br>Successfully identified barriers to PGx adoption that will help implementation of PGx in Singapore mental health settings	Poor response rate may have contributed to selection bias where those who responded to the survey invitation may have differed in their perceptions about PGx versus those who did not respond to the survey invitation<br><br>Only a small number of pharmacists in the sample, which may have not been enough to detect any difference between pharmacist & psychiatrist perceptions<br><br>Questionnaire was developed using various sources from international groups, so may have overlook issues local to Singapore's population

		There were concerns raised about the applicability of PGx research findings to clinical practice			
**Clinician experiences of employing the AmpliChip® CYP450 test in routine psychiatric practice**<br><br>**Dunbar, 2012**	18.5	Uncertainty about how to utilise PGx results & a lack of perceived benefit to using PGx<br><br>Concern over time to completed admin & PGx related tasks, and the length of time to receive PGx results<br><br>Lack of familiarity with PGx amongst staff lead to delays or issues ordering & obtaining results<br><br>Fear that PGx could replace clinical judgement or experience<br><br>Perception PGx was less useful for the 'average' patient & unnecessary if a patient is tolerating treatment<br><br>Concerns about who should pay for PGx & access to PGx tests<br><br>Perceived risk of causing anxiety to the patient by placing more test burden on patients	PGx results can help back up clinical decisions & increase clinician confidence in decisions regarding dosages<br><br>PGx is helpful for those with ADRs or abnormal metaboliser status, and helps validate patients previous medication response<br><br>Knowledge of metaboliser status helped the therapeutic relationship between patient & doctor<br><br>PGx helped to reassure patients about the safety of taking medication – in particular the dose of risperidone to limit ADRs<br><br>Results from PGx tests are useful outside of mental health settings too<br><br>Receiving results electronically improves efficiency but choice of how to obtain results was useful	First study to explore clinician experiences using PGx in a psychiatric care setting<br><br>Findings are useful for future research and implementation of PGx in mental health settings	The study only explored one potential model of using PGx in a psychiatric setting, and is limited to just one PGx test therefore limited generalisability to different models of PGx implementation and different PGx tests that may explore different genes or alleles<br><br>Unknown if the nine clinicians who did not participate in the study hold different beliefs about PGx to those who did participate<br><br>Recall bias could have influenced the study findings<br><br>ROCHE funded the PGx tests and presented to clinicians about PGx prior to ordering which could have influenced their beliefs<br><br>The study may have attracted clinicians with an interest in PGx therefore some potential for bias
**Pharmacogenetic testing in outpatient mental health clinics**	30	Clinician knowledge gap some believe they do not have the knowledge to offer PGx & did	Perception that PGx is a valuable & useful tool, that can help improve tolerability & adherence to medication, help selection & dose of	Comprehensive overview of views towards and experiences of using PGx in outpatient psychiatric clinical care	Those who have utilised PGx in the past may be bias in their viewpoints towards it

**Gainey, 2017**		not receive formal training on how to use PGx in practice<br><br>Concern about the lack of evidence to use PGx –some medication classes more than others, particularly ADHD medicines<br><br>Fear that PGx testing could replace clinical judgement<br><br>Belief that psychiatric PGx reports should contain more information – such as other medicines, drug interactions, ADRs, how to augment treatments, & also herbal/homeopathic medicines<br>Concerns raised about the time involved in waiting for PGx results and the additional paperwork/tasks related PGx<br><br>A lack of education & training available for clinicians to embed PGx within their practice<br><br>Some staff feel PGx reports were too complex & difficult to understand – lengthy PGx reports were off-putting<br><br>Risk of misinterpretation of results – particularly if a traffic-light coding system used in the reports, differing viewpoints of this some believing it oversimplifies the results	medicine, and reassure patients to reduce fear/anxiety about starting medication<br><br>Belief that PGx can validate previous medication experiences particularly if previous treatment resistance or ADRs<br><br>Belief that PGx reports are easy to read – limited need for in house specialist support, but it is helpful when PGx companies that provided access to geneticists & specialists pharmacists to support interpreting PGx results<br>Having colleagues with experience of PGx helped individuals to embed in their practice<br><br>PGx can help understand when a more aggressive/conservative approach to drug dosing should be taken<br><br>Those with previous ADRs or treatment resistance are ideal candidates to receive PGx testing, and useful in those in CAMHS who are treatment naïve or following failure to first line treatments.<br><br>Clinicians reported parents of CAMHS patients felt PGx made treatment safer & reduced ADRs<br><br>Belief PGx is helpful in those who require higher than average doses, have more severe illness or frequency of hospital admissions, & those with autism or are non-verbal	Several novel factors influencing PGx implementation were explored, and many reported findings confirm the findings from previous research	Generalisability of findings may be limited as the study was restricted to just clinics within the geographical region of South Carolina<br><br>There was a limit of professional mix in the study population, so the study findings may not be generalisable to all mental health clinicians, but more specifically nursing mental health clinicians

		Risk of misinterpretation when clinicians are not sufficiently educated and trained to utilise PGx<br><br>Concern over cost of medicines if PGx recommended a medicine that is not off-label<br><br>Ethical concerns about the use & storage of genetic data<br><br>Risk of unrealistic expectations from PGx without sufficient training / PGx counselling<br><br>Belief that PGx is not appropriate for everyone, particularly those stable on medication, medication naïve, or suffering cognitive or personality disorders<br><br>Uncertainty about the impact that PGx can have<br><br>Lack of consensus from staff about the level of information patients should get when receiving PGx counselling<br><br>A lack of educational resources for patients<br><br>Risk of PGx causing psychological distress to patients<br><br>Cost perceived to be a significant factor with many believing it is not cost effective	Perception that PGx positively impacts clinical decisions, & reduce reliance on trial-and-error approach by having a more educated approach & reduce time to find an effective medicine<br><br>Belief PGx can give rationale for prescribing outside of usual practice (e.g., above maximum doses or skipping to 2/3rd line therapies)<br><br>Clinicians felt PGx may help engage patients in shared decision making through open dialogue about pros and cons of treatment<br><br>Perception there is some psychology behind PGx in how it informs patients and makes them feel better about treatment<br><br>Funding to do PGx was perceived to enable its use<br><br>Perception PGx is safe with little medical/physical risks<br><br>Clinicians felt how PGx education is delivered is key to how the patient perceives PGx, and overall, most felt a simple, laymen's terms explanation is better<br><br>Belief that regular updates on results based on new evidence is useful.<br><br>Clinicians reported integration into the EHR, online access and links for		

		Risk of widening health inequalities if insurance only covers PGx testing for specific medicines or populations<br><br>Belief people less likely to order PGx if they have to pay for it themselves	other healthcare providers was useful and beneficial		
**Leveraging the utility of pharmacogenomics in psychiatry through clinical decision support: A focus group study**<br><br>**Goodspeed, 2019**	19	Perception that displaying PGx data in pop ups on a clinical decision support software could lead to user alert fatigue<br><br>Belief that there are issues regarding integration PGx data with a patients medication data in the electronic health record (EHR)<br><br>Scepticism about accuracy of data when incorporating PGx information into a clinical decision support software<br><br>May increase length of appointments due to time constraints for clinicians to identify suitable patients to offer PGx to and to counsel patients about PGx<br><br>Uncertainty about how to update PGx results if new evidence emerges<br><br>Perceived difficulties incorporating PGx into existing clinical pathways – particularly whether results should be	Belief that PGx may be useful in those with poor therapeutic response, experiencing ADRs and for those worried about medication<br><br>Perception that PGx results are useful information to have when conducting an in-depth medication review<br><br>Belief that prescriber trust in the vendor of PGx results is important<br><br>Feeling that PGx results can be used as a prop during discussions with patients and that PGx can help engage patients<br><br>Belief that PGx can be useful for other healthcare providers outside of psychiatry<br><br>Perception that allele visibility within PGx results helps and enables clinicians to trust the PGx vendor<br><br>Acceptance of PGx results being categorised in colour-coded fashion	First study to explore PGx implementation in psychiatry in the context of how PGx data may be incorporated into a clinical decision support system	Generalisability of findings may be limited as all the participants were from one clinical practice in an urban setting, therefore relevance of the findings to non-urban and different clinical practices may be limited<br><br>The study only explored one clinical decision support system – people's experiences and perspectives about PGx data in clinical support software may differ dependent on factors specific to different software programs<br><br>Use of convenience sampling therefore it is unknown if those who did not participate in the study had considerably different views to those who did<br><br>Limited reporting of how the themes were developed following data analysis

		reported on paper or in the EHR.			
**Barriers for Implementation of PGx Testing in Psychiatric Hospitals in Germany: Results of the FACT-PGx Study**<br><br>**Hahn, 2023**	15	Lack of patient awareness about PGx<br><br>Concerns about incorporating additional tasks relating to PGx into existing workflows<br><br>Concern about difficulties shipping PGx samples to laboratories<br><br>Raised concerns about data protection & privacy relating to PGx<br><br>Patients worried about the consequences from PGx test findings, including if it can be used for diagnostic or prognostic purposes.<br><br>Concern about the turnaround time for PGx testing and time it takes to receive the results back to the user	Positive overall attitudes towards PGx, with majority interested to find out more about PGx<br><br>Belief that PGx may reduce hospital admissions<br><br>Those with more negative experiences of antidepressants, or with a higher educational attainment were more likely to perceive PGx to be potentially beneficial to them<br><br>Majority were willing to pay for PGx, those with a higher educational attainment were willing to pay more<br><br>A period of approximately two weeks was deemed to be an acceptable timeframe to wait for results, those without previous experience of antidepressants	First study of its kind to explore patient perspectives towards PGx testing in psychiatry in Germany<br><br>Demonstrated those with a higher educational attainment are more willing to pay for PGx	Potential bias introduced into the study by asking about cost to participants who had received free PGx testing as part of a research study so may not be representative views<br><br>Study did not explore whether PGx had influenced treatment decisions, and this could have influenced attitudes towards PGx and answers in the survey
**Psychiatrists' attitudes regarding genetic testing and patient safeguards: A preliminary study**<br><br>**Hoop, 2008**	21	Belief that PGx should only be offered if the psychiatrist believes the benefits outweigh the risks<br><br>Belief that psychiatrists should be competent in their understanding of PGx and interpreting PGx results before offering PGx to patients	Many felt that PGx is relevant to a psychiatrist's role<br><br>Belief that introduction of PGx into clinical practice would dramatically change prescribing practices<br><br>Perception that PGx can be useful to identify those are high or low risk of ADRs, and in determining the dose of psychotropic medicines	First study to explore psychiatrist perspectives towards PGx testing<br><br>Participants were recruited nationally represented a diverse mix of geographical locations	Lack of inclusion of other healthcare professionals<br><br>Limited diversity in the sample population with low numbers of Hispanic responders and no Black or African participants<br><br>Risk of non-response bias in which those who did respond may have significantly different views to those who did not respond

		Perception that pre and post PGx counselling should be provided to patients<br><br>Belief from some there are psychosocial risks associated with PGx<br><br>Perception there is a lack of legal framework or oversight regarding the uptake of PGx in practice	Belief that consent should be obtained prior to conducting PGx and that PGx results should be kept confidential		Quantitative study design may have limited scope to explore psychiatrist attitudes
**Clinical and ethical considerations in pharmacogenetic testing: Views of physicians in 3 "early adopting" departments of psychiatry**<br><br>**Hoop, 2010**	23	Most had received no or minimal PGx training<br><br>Females perceived there are more risks associated with PGx than males<br><br>Perceived risks associated with PGx including a risk of psychological distress, and impact on patient employability or insurability<br><br>Concern that secondary information about disease susceptibility being discovered by PGx testing<br><br>Issues raised about obtaining patient consent<br><br>Indecision over whether PGx will be too expensive for patients<br><br>Perceived lack of peer-reviewed evidence to support PGx in reducing ADRs or	Optimism for the use of PGx<br><br>Belief PGx is a new & useful laboratory tool<br><br>Belief that PGx will dramatically change psychiatric practice<br><br>Agreement that PGx should be offered by psychiatrists in appropriate clinical scenarios – those with more PGx training agreed more strongly with this<br><br>Those with more PGx training felt more competent in tasks relating to the use of PGx<br><br>Those with more experience of using PGx felt more confident in identifying clinical scenarios to use PGx<br><br>Agreement that PGx is more useful when there is medication intolerance & treatment resistance	First study to explore perspectives towards using PGx in psychiatry using mixed methods	Findings may not be generalisable to all psychiatry departments because there may be features of early adopting departments that make them considerably different from other psychiatry departments<br><br>Possible there is self-selection bias from those who volunteered to participate<br><br>Potential recall bias played a role in psychiatrist response in the study

		improving the chance of a therapeutic response<br><br>Belief that there is a long way to go before PGx is in widespread use<br><br>Concern about the time & cost involved in PGx<br><br>Belief there is a lack of pre-test counselling about PGx & managing their expectations<br><br>Belief that the benefits of PGx may be overstated	Belief that other than bloods being taken there are no identifiable risks to using PGx<br><br>Belief that to ensure PGx is ethical it should be confidential, obtain patient consent, and include pre- and post-test counselling		
**PGx in Psychiatry: Patients' knowledge, interest, and uncertainty management preferences in the context of pharmacogenomic testing**<br><br>**Kastrinos, 2020**	26	Patients had low familiarity with what PGx is	Strong interest in the use of PGx from patients with mental health conditions<br><br>Interest in the use of genetic testing in psychiatry was a positive predictor of PGx interest<br><br>Belief that PGx can reduce uncertainty about treatment or illness<br><br>Patients had a preference to seek information about PGx rather than avoid & this was a positive predictor of their interest in PGx	First study to use underpinning theory of uncertainty management theory in the context of PGx use in psychiatry<br><br>One of the first studies to quantitatively explore patient perspectives towards PGx in psychiatry	Sampling method may have led to a sample that is not representative of psychiatry patients, as those who volunteered are more likely to be 'information seeking' as they are more informed and actively involved in their health care<br><br>The sample population also lacked diversity, as participants were more likely to be well educated, white and female so generalisability of study findings may be limited<br><br>Study did not appear to consider if participants had received PGx testing which may have influenced their responses
**Patients and Clinicians Report Higher-Than-Average Satisfaction with Psychiatric Genotyping for Depressed Inpatients**	9	Most patients were unaware of PGx prior to involvement in the study	Clinicians perceived a table that included PGx compatible medicines in the PGx results report to be useful & made them more likely to utilise the results	Evaluated the use of PGx testing in a real-life clinical practice setting<br><br>First study to include both patients and clinicians	Lacking explanation of data analysis so it is unclear how they controlled for bias

**Kung, 2011**		Delay in receiving results meant that in some instances the PGx results were not available to the clinician prior to the patient being discharged – leading to lower satisfaction rates	Clinicians overall reported satisfaction with the use of PGx<br><br>Patients believed that clinicians explained what PGx is well<br><br>Patients had a higher-than-average satisfaction with the use of PGx<br><br>A quicker turnaround time in receiving the PGx results led to an increased chance of the PGx results being utilised		There is no information about the sample characteristics (both patient & clinician) therefore difficult to draw conclusions about how generalisable the findings are
**Acceptability of pharmacogenetic testing among French psychiatrists, a national survey**<br><br>**Laplace, 2021**	28.5	Perception that PGx was not easy to use<br><br>Perception there is a lack of public information about PGx availability<br><br>Belief that professional guidance for PGx recommendations is unclear<br><br>Belief among most psychiatrists that they lacked knowledge about PGx & did not feel informed enough to identify situations to order PGx<br><br>Cost was perceived to be a risk with PGx<br><br>There was delay in receiving some PGx results<br><br>Most psychiatrists had not received training about PGx and perceived themselves to	Overall acceptability towards PGx was intermediate but higher than average<br><br>High perceived usefulness of PGx<br><br>Belief that PGx can improve psychotropic response both in terms of efficacy & tolerability<br><br>Perception that PGx can reduce the time to find the most appropriate treatment<br><br>Enthusiasm among psychiatrists to learn more about PGx<br><br>Belief that PGx is reliable<br><br>Belief that PGx will become a common part of clinical practice<br><br>Psychiatrists more favourable towards using PGx in treatment resistant depression or	Used theories and models of acceptability as a framework through which to explore the study aims<br><br>Adequate sample size that met the study power calculation for study sample<br><br>Used appropriate statistical tests to measure effects of sample characteristics and given responses	Potential selection bias due to the survey being online and therefore sample may include those more likely to engage with online surveys<br><br>Associations between sample characteristics and response are associations and caution advised not to overstate these findings as they are not causal relationships<br><br>Specific aspects about PGx were not explored including their perspectives towards which type of test (monogenic vs polygenic) should be adopted, what information they desire from a test, and level of satisfaction following using PGx testing

		lack confidence to explain the pros/cons of PGxT or adjust their clinical decisions based on PGx results<br><br>Perceived ethical concerns about the misuse of PGx data and incidental findings from PGx data<br><br>Those with a more psychosocial approach versus a biological approach to psychiatry were less likely to use PGx<br><br>Belief that PGx could negatively impact the prescriber-patient therapeutic relationship<br><br>Psychiatrists had little reported experience using PGx<br><br>Concern about the psychological impact PGx may have on patients	schizophrenia, than in bipolar disorder<br><br>Most psychiatrists perceived the benefits of PGx to outweigh the risks in the context of treatment resistant depression<br><br>Majority of respondents would accept a PGx test themselves		
**Patients' Perspectives on Psychiatric Pharmacogenetic Testing**<br><br>**Liko, 2020**	23	Perception of uncertainty about whether PGx can be helpful<br><br>Belief that PGx results did not give clear guidance on which medication to choose<br><br>Perception that PGx costs too much for the value gained<br><br>Some believed that results that used the traffic light system	Pre PGx test counselling perceived to help avoid misinterpretation of results<br><br>Belief that PGx is indicated in the event of medication failure due to inefficacy or ADRs occurring<br><br>Experience of having PGx testing enables patient understanding & awareness of what PGx is	Study allowed for pre- and post-test evaluation of perspectives about PGx testing providing useful findings<br><br>First study to adopt semi-structured interviews with patients offering an in-depth insight into patient views and perspectives	Potential that recall bias could have impacted the study findings because the mean time between having a PGx test and participating in an interview was over a year<br><br>Convenience sampling adopted therefore a risk that those patients selected for interviews differed from those who were not selected in a significant way and may have had different viewpoints or perspectives

		were unclear and risked misinterpretation	Belief that PGx is a useful tool to help guide prescribing and help clinicians choose medication to prescribe<br><br>Perception that changes to prescribed medicine based on PGx led to improved response<br><br>Belief among patients prior to PGx testing that it can help find the right medicine for them<br><br>Belief that PGx results helped to validate previous response to medicines<br><br>Perception that in those who have had multiple medication failures the benefit of PGx would outweigh the cost of PGx		Lack of diversity in the study sample as they were all defined as Caucasian<br><br>Study conducted at a specialist depression centre where patients are more likely to be treatment resistant therefore generalisability to patients in other mental health settings is limited
**Attitudes on pharmacogenetic testing in psychiatric patients with treatment-resistant depression**<br><br>**McCarthy, 2020**	22	Patients had some concern about the impact of receiving PGx results could have on their family members<br><br>Some patients highlighted concern about the risk of discrimination based on PGx results – this was more of a concern for non-Caucasians	Overall favourable opinions towards the use of PGx testing to help select medication<br><br>Belief that PGx can help identify those at risk of ADRs or poor therapeutic response<br><br>Viewpoints that PGx helped people understand their illness<br><br>Perception that patients would be able to cope emotionally with receiving PGx results, despite being depressed and that level of depression did not impact upon their capacity to cope	Study confirmed findings from previous research about patient viewpoints towards PGx<br><br>Novel finding that level of depression did not appear to influence ability to cope with finding out PGx results	Study population was not representative as was largely Caucasian, male and Veteran (so unemployed or retired) therefore findings may not be generalisable to all depressed populations<br><br>The study did not follow up post PGx testing perceptions about PGx<br><br>Instrument used to collect data was adapted from a study exploring PGx in cancer care therefore may not have been completely appropriate for use in context of PGx in depression

			Overall little concern about being discriminated against based on PGx results		Risk of selection bias as participants had already agreed to take part in a clinical PGx study
**The Influence of Genotype Information on Psychiatrists' Treatment Recommendations: More Experienced Clinicians Know Better What to Ignore**<br><br>**McMichael, 2017**	13.5	Genotype information did not significantly influence psychiatrists decisions in the discrete choice experiment<br><br>Older or more experienced psychiatrists were less likely to consider the genotype information when making treatment decisions<br><br>Psychiatrists with more than 15 years clinical practice were likely to almost not consider genotype information at all	Younger psychiatrists were more likely to consider genotype information when prescribing treatment in schizophrenia or psychosis<br><br>Those psychiatrists with less than a year of clinical experience were 50% likely to use genotype information when making decisions	First study of its kind to explore whether psychiatrist experience influences whether they use genotype information when prescribing	Case study design was flawed as the content within case study was able to manipulate or effect the respondents choice in the discrete choice experiment<br><br>Lack of transparency about the sampling and recruitment process used<br><br>Considered genotype information in context of already knowing a patient's response, yet often this is unknown at the point of prescribing
**Anticipated outcomes from introduction of 5-HTTLPR genotyping for depressed patients: An expert Delphi analysis**<br><br>**Oestergaard, 2010**	19.5	Belief that serotonin transporter genotyping would not be available to most patients in primary care<br><br>Perception there is a lack of data or evidence about the impact of 5-HTTLPR genotyping has on relapse or recurrence rates of depression	Belief that 5-HTTLPR genotyping would be useful in certain special situations<br><br>Perception that 5-HTTLPR genotyping to help guide antidepressant prescribing would more likely be used in secondary care than primary care<br><br>Genotype information viewed as a patient characteristic along with other patient characteristics that can be considered during prescribing	First study to adopt Delphi methodology in context of PGx use in psychotropic prescribing and reported similar sample size to previous research using Delphi methodology	Role of participants in clinical practice was unclear<br><br>Some wording in the questions asked as part of the Delphi methodology was ambiguous and participants may have been unclear about what these terms were intended to mean, potentially leading to differences in the way different participants interpreted the questions
**Best-worst scaling methodology to evaluate constructs of the Consolidated Framework for Implementation Research: application to the**	23.5		Identifying PGx champions was seen as an important construct of the Consolidated Framework for Implementation Research (CFIR) to help facilitate PGx use for antidepressants	First study to use best-worst scaling methodology to assess factors influencing PGx implementation which is a simple effective way to determine what	The sample size of the study was small limited to the members of the IGNITE network<br><br>Unclear from the study which members of the participating

**implementation of pharmacogenetic testing for antidepressant therapy**<br><br>**Salloum, 2022**			Leadership engagement was also determined to be an important factor to help PGx implementation<br><br>Knowledge and beliefs about PGx were perceived to be an important factor to facilitate the implementation of PGx<br><br>PGx evidence strength & quality was believed to be an important factor influencing PGx implementation for antidepressants	people perceive to be important factors<br><br>The study successfully assessed the factors influencing PGx implementation at several sites	organisations completed the study, who completed the survey may have influenced the responses of specific organisations.
**Psychiatric pharmacists' perception on the use of pharmacogenomic testing in the mental health population**<br><br>**Shishko, 2015**	21	Many psychiatric pharmacists perceived themselves to be less knowledgeable about PGx<br><br>Among psychiatric pharmacists there was mixed awareness about what PGx is<br><br>Perception amongst some that PGx lacks clinical benefit<br><br>Cost was perceived to be a barrier to using PGx, with few psychiatric pharmacists believing PGx to be a cost-effective intervention	Psychiatric pharmacists believed there are a range of potential benefits to using PGx including helping to guide medication selection, reduce ADRs, and reduce long-term healthcare costs<br><br>Belief that PGx can help reduce treatment resistance<br><br>Perception that PGx can help improve evidence-based medicine in mental health<br><br>Belief that PGx can help to improve a patient's quality of life	First study to explore the views of specifically pharmacists about the uptake of pharmacogenomics	Study sample may not be representative as inclusion was limited to members of the CPNP, other pharmacists in the same workplace that are not members of the CPNP may differ in their views.<br><br>Some ambiguous questions that were unclear and could have been misinterpreted by the participant. Generally, questions were limited in scope and could have explored specific factors in more detail
**Assessing the impact of pre-test education on patient knowledge, perceptions, and expectations of pharmacogenomic testing to guide antidepressant use**<br><br>**Sloat, 2022**	28	Those less knowledgeable about PGx were less likely to want to pursue PGx testing<br><br>Patients who had trialled more than one antidepressant were less likely to want to pursue PGx testing	Education about PGx facilitated more positive views towards having PGx testing, more interest in PGx and enabled patients to feel more informed about PGx testing<br><br>Intention to have PGx testing was correlated to perceived ease of PGx	First study of its kind to demonstrate an educational resource is an effective intervention to improve understanding & awareness of PGx in the context of antidepressants, and will inform future research exploring PGx	Study relied on self-assessment of depression which was not confirmed or validated.<br><br>Generalisability of the study may be limited due to the study sample being young & educated which may not be representative of wider

			testing and social norm of having PGx testing	education for patients in mental health<br><br>Also demonstrated education has the greatest impact on treatment naïve patients, a novel finding	populations of people living with depression<br><br>Did not some factors that are known to effect treatment effectiveness such as access to healthcare & medicines adherence
**Pharmacogenomic Testing for Major Depression: A Qualitative Study of the Perceptions of People with Lived Experience and Professional Stakeholders**<br><br>**Slomp, 2022**	25.5	Clinicians worried about the cost of PGx<br><br>Concerns about whether PGx will impact medicines adherence<br><br>Ethical issues raised around the use and privacy of genetic data – some patients concerned about the secondary use of genetic data which would act as a barrier to having PGx testing<br><br>Concern about risk of discrimination based on PGx results, especially from insurance companies, and discrimination based on ethnicity<br><br>Vulnerability of patients & risk of worsening mental state / emotional distress raised as barriers to PGx use<br><br>HCP lack of knowledge & expertise viewed as a potential issue when interpreting PGx results	Perception that PGx has a range of potential benefits including reducing ADRs, reduce need for polypharmacy, reduce time to find an effective medication, validate previous medication experiences, reduce healthcare costs, and reduce fear/hesitancy to starting medication<br><br>Belief PGx can help destigmatise mental health conditions<br><br>Agreement that PGx could help reduce the trial-and-error approach to prescribing antidepressants and achieve a faster response to antidepressants<br><br>Perception among some professionals that PGx can be cost effective<br><br>Belief PGx may help improve medication adherence to antidepressants<br><br>Belief that PGx can increase prescriber confidence in clinical decisions, and may improve patient-prescriber relationship and trust	The study was the first of its kind to explore both the perspectives of people with lived experience and professionals in relation to PGx use in depression	The sample was not representative or diverse, as participants were limited to those who had computer access and that were engaged in social media therefore were not representative of the wider population with depression in British Colombia and the sample was largely white in ethnicity

		Potential risk PGx may give patients false hope regarding treatment & recovery, poses a risk of distress which would act as a barrier to PGx use<br><br>Turnaround time for PGx results or delay in prescribing could act as a barrier to use<br><br>Concern about how results would be updated based on new evidence or re-analysis of existing evidence<br><br>PGx testing could contribute to prescribers becoming over reliant on medicines as intervention rather than non-pharmacological treatments<br><br>Perception that PGx may add to already existing issues in mental healthcare regarding clinician time, accessing to psychiatric care, & stigma of mental health illness, and how PGx may fall victim to these issues			
**Psychiatrist attitudes towards pharmacogenetic testing, direct-to-consumer genetic testing, and integrating genetic counselling into psychiatric patient care**<br><br>**Thompson, 2015**	17.5	Uncertainty amongst psychiatrists if they would offer PGx<br><br>Psychiatrists had uncertainty about whether they believed PGx could improve treatment decisions<br><br>Psychiatrists were generally not familiar with genetic	Belief among psychiatrists that PGx can help both patients and clinicians to make decisions about treatment<br><br>Perception that PGx will become a standard part of prescribing in psychiatry practice in the future<br><br>Belief from most psychiatrists included that the help of a genetic counsellor to discuss PGx results	First study to explore psychiatrists views towards collaboration with genetic counsellors in context of PGx<br><br>Adds to the literature about psychiatrist perspectives towards using PGx	Survey/questionnaire was not piloted therefore quality of instrument may have been low<br><br>Meaning of different terms included in the survey were not explained<br><br>Sampling method was limited by selection bias, as many of the respondents were involved in a

		counselling or use of genetic counsellors	with patient and/or clinician would be helpful		clinical PGx study, therefore potential bias as these respondents' responses may have differed to psychiatrists not involved in the clinical PGx study<br><br>Generalisability of the results may be limited, as most of the respondents were from UCSF research institutes that are possibly not representative of all psychiatrists
**Multisite evaluation of institutional processes and implementation determinants for pharmacogenetic testing to guide antidepressant therapy**<br><br>**Tuteja, 2022**	21.5	There were distinctions between different sites in what they deemed to be the most important factors that determined successful implementation of PGx to support antidepressant prescribing	Evidence strength & quality, and provider knowledge were perceived to be must important factors for implementing PGx<br><br>A local PGx champion and leadership engagement were believed to be a factor helping the implementation of PGx<br><br>Multiple referral routes were perceived to help use of PGx, as well as professionals collaborating in a multidisciplinary fashion<br><br>Use of electronic health records (EHR) to support PGx tasks was believed to help implementation and use of PGx<br><br>Patient needs & resources, and external policy & incentives were also perceived to be important factors outside the intervention that determined how PGx implementation success	The results expanded on previous findings in the field of PGx implementation science and helps understand the barriers/facilitators to PGx implementation	Findings from the study may not be representative on organisations that are not members of the IGNITE network, therefore the generalisability of findings may be limited<br><br>Many of the included organisations in the study had a dedicated precision medicine or PGx service that helped oversee implementation of PGx which may not be possible for all mental health care services wanting to adopt PGx<br><br>The use of BWS is limited by what people perceive to be important, which is possibly different to what is actually important in practice.<br><br>BWS is also limited as there may be factors not considered with BWS methodology that are important for PGx implementation but not explored in this study

**Pharmacogenetics in Psychiatry: Perceived Value and Opinions in a Chilean Sample of Practitioners**<br><br>**Undurraga, 2021**	17	Perceived lack of awareness about PGx in psychiatry<br><br>Cost was perceived to be an issue as practitioners felt the current cost was too high<br><br>Reported lack of availability of PGx services<br><br>Belief that prescribers themselves had a lack of knowledge about PGx<br><br>Practitioners believed there was a lack of clinical utility to support PGx use in psychiatry<br><br>Concern about the translation of international PGx guidelines to Latino-American populations	Perception that PGx can help to reduce ADRs or explain previous experience of ADRs<br><br>Belief that PGx can help predict drug response<br><br>Belief among practitioners that PGx will be integrated into clinical practice by 2025	First study to explore Chilean psychiatrist perspectives towards PGx<br><br>Findings support the outcomes from similar research conducted in other countries	Sampling method limits the finding of the study as recruited participants were primarily from the Santiago area which is possibly not representative of all areas of Chile<br><br>Only a small proportion of participants had used PGx, of which the vast majority were private healthcare providers which could have introduced bias into the study if their views differ considerably from those who have not used PGx in public healthcare
**Primary care and mental health providers' perceptions of implementation of pharmacogenetics testing for depression prescribing**<br><br>**Vest, 2020**	25	Concern about the risk of misinterpretation of PGx results, especially if colour coding is used to report PGx test results<br><br>Limited awareness or knowledge of the evidence supporting the use of PGx<br><br>Belief that the current evidence supporting clinical evidence of PGx is not definitive<br><br>Perception that PGx should not be used or should be used cautiously until more evidence to support its use is available	Belief that PGx is simple to use<br><br>Perception that PGx provides an additional piece of information to help inform prescribing decisions<br><br>Belief that PGx can help those with previous treatment failure<br><br>Hope that PGx will become better than the current trial-and-error approach to prescribing<br><br>Desire from professionals to trial PGx use in practice and tailor to patient characteristics based on experience	Use of online focus groups allowed for the same researchers to facilitate the focus groups which reduced the impact variation in interviewer bias could have on the study findings	General belief PGx is a multidisciplinary intervention but this study failed to include non-medical professionals in the focus group therefore was narrow in the applicability of findings<br><br>A presentation was provided to participants prior to participation in focus groups which could have influenced view provided by those who took part<br><br>Many participants were already taking part in the PRIME (PGx clinical study) which could have influenced their views and meant their views are significantly

		Concerns about how PGx is integrated into existing clinical pathways<br><br>Belief that delays in prescribing while waiting for PGx results to be returned could be problematic<br><br>Concern from professionals about being able to counsel or educate patients about PGx – particularly finding the time to do so<br><br>Primary care providers believed there would be concerns about how to fit PGx into standard practice			different from those not involved in the trial, therefore, the results may not be generalisable to all primary care and mental health providers<br><br>Only explored selected constructs of the CFIR – other elements of CFIR may be equally important factors
**Physicians' opinions following pharmacogenetic testing for psychotropic medication**<br><br>**Walden, 2015**	12	Levels of understanding of PGx results differed by sex – females had a lower reported perceived understanding of PGx results	Those who had used PGx results in their practice were generally satisfied with the information they received<br><br>Perception that PGx is useful in helping to prescribe medications<br><br>Agreement that PGx will become a standard part of clinical practice in the future<br><br>Majority would recommend patients to future PGx studies	The first study to explore viewpoints towards PGx for psychotropic medicines, specifically following the use of PGx (rather than exploratory investigation of views prior to using PGx in practice)	Participants were primarily from academic hospitals and already taking part in a clinical PGx study which may have influenced their views towards PGx and cause bias in the findings<br><br>The generalisability of the findings are limited and may be only relevant in the context of the specific model of PGx that was adopted, and may not represent views towards different possible models of integrating PGx in clinical practice
**Perspectives of a pharmacist-run pharmacogenomic service for depression in**	26	Feeling from prescribers & pharmacists that additional education & training is needed before implementing a new	Collaboration between a doctor & trained PGx pharmacist facilitates the uptake & use of a PGx depression service	Demonstrated a collaborative approach to PGx for antidepressant prescribing in primary care can yield positive	Generalisability of the findings may be limited because participants were selected using purposive

**interdisciplinary family medicine practices**<br><br>**Weinstein, 2020**		PGx service – both at the basic undergraduate level but also more specialised for use in depression<br><br>Belief that patient education about pros & cons of PGx is essential<br><br>Identification of suitable patients to receive PGx is an important factor to implementing PGx	Belief that pharmacists can help with interpretation of PGx results and help selecting a suitable medicine<br><br>Perception that a pharmacist led PGx clinic is appropriate to slot into existing clinical pathways<br><br>Belief that a team-based approach, with open communication and trust helps implementation of a PGx clinic<br><br>An interdisciplinary approach to monitoring & follow-up of PGx believed to help implementation<br><br>Belief among doctors that PGx can help reduce trial-and-error approach to prescribing<br><br>Belief that PGx is a tool that can help inform selection of initially prescribed antidepressant and will become a routine part of practice<br><br>Belief that a short delay in prescribing while waiting for PGx results would be appropriate for most patients<br><br>Among pharmacists there is belief that PGx presents opportunities to expand the role of pharmacists and help embed pharmacists in primary care	perceptions from pharmacists and prescribers	sampling from a single community teaching hospital<br><br>The study adopted one model of PGx implementation therefore findings may not be generalisable to all models of PGx adoption

			Prescribers perceived pharmacists to be vital to the success of a PGx service		
**Patient Attitudes Toward PGx Testing in Psychiatric Treatment**<br><br>**Tamaeiev, 2023**	16.5	Most patients had never heard of PGx<br><br>Perception among patients that PGx should not be offered to all patients<br><br>Cost was perceived to be a risk or concern related to the use of PGx, and whether health insurance or employer would cover this cost<br><br>Males were less likely to perceive PGx positively compared to females<br><br>Some concern about the privacy of genetic information determined by PGx testing	Perception among patients that they could benefit from having PGx testing – more so in those who had heard of PGx previously<br><br>Belief that PGx can be used to help doctors make treatment decisions<br><br>Perception among patients that doctors would be able to interpret PGx results<br><br>Desire from most patients to retain a copy of their PGx results<br><br>Belief that patients would feel more confident in taking their medication if they had obtained PGx testing<br><br>Stronger perception among inpatients, over outpatients, that PGx would help patients feel more confidence in their doctors treatment decisions – possibly due to less satisfaction in terms of helpfulness of current medication<br><br>Patients reported to be happy to discuss their PGx results with a genetic counsellor<br><br>Willingness to undergo PGx testing	Corroborates findings from previous research exploring patient attitudes towards PGx testing	Although inpatients & outpatient was a relatively small sample of inpatients, many of which had already consented to participating in other research studies so may be more inclined to seek novel treatment approaches<br><br>All of the sample was recruited from a single hospital therefore generalisability of the results was limited<br><br>Items were added to the survey later in the data collection phase, therefore had limited sample to review the answers to new items<br><br>Limitations to use of a Likert scale, in particular distinguishing between 'not sure' and 'maybe' options
